# Denosumab treatment of osteoporotic women arrests cortical bone remodeling events at the reversal-resorption phase but does not affect ongoing bone formation

**DOI:** 10.1093/jbmrpl/ziaf181

**Published:** 2025-11-22

**Authors:** Xenia Goldberg Dahl, Jean-Paul Roux, Jean-Marie Delaisse, Shuang Huang, Pascale Chavassieux, Christina M Andreasen, Thomas L Andersen

**Affiliations:** Molecular Bone Histology Team, Research Unit of Pathology, Department of Clinical Research, University of Southern Denmark, 5230 Odense M, Denmark; Univ Lyon, INSERM, UMR 1033, UFR de Médecine Lyon-Est, F-69008 Lyon, France; Molecular Bone Histology Team, Research Unit of Pathology, Department of Clinical Research, University of Southern Denmark, 5230 Odense M, Denmark; Center for Design and Analysis, Amgen Inc., Thousand Oaks, CA 91320, United States; Univ Lyon, INSERM, UMR 1033, UFR de Médecine Lyon-Est, F-69008 Lyon, France; Molecular Bone Histology Team, Research Unit of Pathology, Department of Clinical Research, University of Southern Denmark, 5230 Odense M, Denmark; Department of Pathology, Odense University Hospital, 5000 Odense, Denmark; Molecular Bone Histology Team, Research Unit of Pathology, Department of Clinical Research, University of Southern Denmark, 5230 Odense M, Denmark; Department of Pathology, Odense University Hospital, 5000 Odense, Denmark; Department of Forensic Medicine, Aarhus University, 8200 Aarhus N, Denmark

**Keywords:** bone modeling and remodeling, osteoporosis, antiresorptives, osteoblasts, osteoclasts

## Abstract

Denosumab is an anti-resorptive therapy that effectively reduces fracture risk and increases BMD in women with postmenopausal osteoporosis. In this study, we focused on the less well-investigated cortical remodeling process and its transition from erosion to formation after denosumab treatment, using a histomorphometric classification of intracortical pores. Cortical bone is more prominent in non-vertebral bones, where denosumab is less effective in fracture reduction. In iliac crest biopsies from the FREEDOM-study, where postmenopausal osteoporotic women were treated with placebo (*n* = 43) or denosumab (*n* = 43) for 2-3 yr, the cortical microstructure and remodeling stage and type of intracortical pores were analyzed. Cortical thickness was unaffected in the denosumab group vs placebo (*P* = .9). Mean pore diameter was significantly decreased in the denosumab group vs placebo (*P* < .002) with no change in the number of pores per tissue area (*P* = .83). However, the cortical porosity was not decreased in the denosumab vs placebo group (*P* < .077). When stratifying the pores according to their remodeling stage, the eroded pores had an increased contribution to the total pore area in the denosumab vs placebo group (*P* = .001). Eroded-formative and formative pores had a decreased contribution to the total pore area (*P* < .001). The contribution of quiescent pores was unchanged. The increased contribution of eroded pores and the concomitant reduced contribution of eroded-formative and formative pores indicate that the transition from erosion to formation is largely limited, while pores with ongoing bone formation at the time of initiation of denosumab treatment are refilled.

## Introduction

Osteoporosis is a skeletal disease characterized by low bone mass, leading to increased bone fragility and fracture risk.^1^ Despite intensive research and several therapeutic agents being available for treatment of osteoporosis, long-term treatment with continued effects and without increasing the risk of adverse effects remains a challenge.^2^^,^^3^ Furthermore, the available treatments differ in their ability to reduce vertebral and non-vertebral fractures—with most therapies being superior in the prevention of vertebral fractures.^4^ This discrepancy between skeletal sites might be due to strain-differences, the local concentration of the drug at different sites, and different ratios of trabecular vs cortical bone. The most used osteoporotic treatments are anti-resorptives. Anti-resorptive treatment reduces bone resorption but consequently, the bone formation is reduced too, as bone formation is strictly coupled to bone resorption during the remodeling process. This study focuses on how denosumab treatment of osteoporosis affects the transition from resorption to formation during the cortical bone remodeling process.

In physiological adult bone remodeling, the coupling and balance between bone formation and bone resorption ensures a constant bone mass. The bone loss in aging and osteoporosis is the consequence of an insufficient coupling-mediated transition from resorption to formation, where a delayed initiation of bone formation causes a prolonged/arrested reversal-resorption phase at the level of the individual BMU also called bone remodeling units (BRU).^5–7^ During the reversal-resorption phase, the eroded bone surface is prepared for bone formation by colonizing osteoprogenitors (osteoblastic reversal cells) intermixed with osteoclasts that deepen the erosion. Another important action during the reversal-resorption phase is the recruitment of additional osteoprogenitors, which gradually differentiate into mature bone-forming osteoblasts that initiate bone formation once a critical density is reached.^6^^,^^8–10^

Treatment of postmenopausal osteoporotic women with anti-resorptives increases BMD and reduces fracture risk. The increase in BMD differs depending on the type of anti-resorptive. The bisphosphonate alendronate initially increases BMD in the hip until it reaches a plateau,^11^ whereas hip-BMD continues to increase with the anti-RANKL-antibody denosumab. Despite the difference in BMD-gain between alendronate and denosumab, both types of anti-resorptives induce a reduction in serum bone formation markers,^12–14^ suggesting different effects on the coupled bone resorption and formation.

The cortical effects of denosumab after 1 yr have been investigated using HR-pQCT, reporting decreased cortical porosity in the proximal femur after 1 yr of treatment.^15^ In iliac crest biopsies from the FREEDOM and STAND studies, μCT showed decreased porosity after 2 yr of treatment but not after 3.^16^ Histomorphometric analyses of iliac crest biopsies from the FREEDOM study failed to demonstrate decreased cortical porosity after both 2 and 3 yr of denosumab treatment.^17^ In this study, we investigate the cortical effects after 2-3 yr of denosumab treatment.

From a bone formation standpoint, histomorphometric analyses from the FREEDOM study demonstrated significant reductions in mineralizing surfaces across the trabecular, periosteal, endosteal, and intracortical compartments in iliac crest biopsies from denosumab vs placebo treated patients.^16^^,^^17^ The lack of mineralizing surfaces in the denosumab treated patients hampered any proper analysis of the mineral apposition rate, reflecting the rate of mineralization once initiated.^16^^,^^17^ None of these studies addressed whether the reduced bone formation was only the result of a reduced activation of resorption events, or also the result of an insufficient transition from erosion to formation during the remodeling process. Interestingly, resorption independent modeling-based bone formation has been reported to persist upon denosumab treatment in the periosteal surfaces of femoral bone in monkeys,^18^ and in 4 human femur necks.^19^

The cortical compartment is ideal for the investigation of bone remodeling dynamics and the impact of denosumab on this process, since bone loss can be quantified through porosity in the intracortical bone. Furthermore, each intracortical pore represents a bone remodeling event in which pore size can be related to specific bone surface events (such as resorption). In that way, a relation between the phases of bone remodeling and bone loss can be established. The interest of this study is to establish such a relation, by classifying the pores according to whether they show erosion, formation, erosion, and formation or quiescence.

In this study, a new histomorphometric classification of intracortical pores^20^ was applied to sections from iliac crest biopsies obtained from the FREEDOM study. This allowed us to investigate the hypothesis that treatment with denosumab affects the cortical bone microstructure as well as the intracortical and periosteal remodeling events ability to transition from bone resorption to formation, causing an arrest of the remodeling events in the reversal-resorption phase.

## Material and methods

### Clinical trial

This study was conducted on biopsies from the randomized, double-blinded, placebo-controlled phase 3 FREEDOM trial, where 7868 postmenopausal osteoporotic women were randomly assigned to denosumab (60 mg every 6 mo), or placebo for 3 yr. Inclusion and randomization criteria have been described previously.^17^^,^^21^ Briefly, patients were eligible for enrollment if they were 60-90 yr and had a T-score between −2.5 and −4.0. Exclusion criteria included the presence of conditions affecting bone metabolism other than osteoporosis, or previous long-term treatment with bisphosphonates or within the last 12 mo. A sub-study included patients from the placebo group (*n* = 45) and the denosumab group (*n* = 47), who had trans iliac biopsies taken with a 7.5 mm inner diameter trephine after 2 or 3 yr of treatment.^16^

For this study, biopsies previously used for classical histomorphometric analysis (patients treated with placebo *n* = 45 and denosumab *n* = 45) were available.^17^ We were able to include 43 patients treated with placebo and 43 patients treated with denosumab, excluding 4 patients due to biopsy/section quality issues. These issues included a lack of cortical bone in the mounted section (*n* = 1), cortical bone that was too bulky/destroyed to be clearly visualized (*n* = 2), and cortical bone sectioned at a wrong angle making it difficult to delineate the cortex and its pores (*n* = 1). This study mainly reports the pooled effect after 2 and 3 yr of denosumab vs placebo treatment. Additionally, 24 of the included patients had biopsies taken after both 2 and 3 yr. In the main data, their 2-yr biopsies were excluded, while the paired effects from 2 to 3 yr (placebo *n* = 16, denosumab *n* = 6) of treatment are reported in Table S1.

### Preparation of biopsies

The undecalcified trans iliac bone biopsies were embedded in glycol methacrylate, and cut in 8-μm-thick sections, which were stained with Goldners Trichrome, as described.^17^ In this study, stained sections were imaged on a VS200 slide scanner (Olympus, Denmark, Ballerup) under polarized light in Z-stack mode. Obtained stacks were combined into a single-plane image adjusted to optimal focus throughout each biopsy.

### Histomorphometry—cortical microstructure

In each cortex, all intracortical pores were identified and measured using VS200 Desktop 3.2.1 & DNN (Olympus, Denmark, Ballerup) utilizing trained deep neuronal networks. The identification and measurement of pores were semi-automated with a manual check and corrections performed on each biopsy (Figure S1). For all identified pores, their respective pore area, perimeter, and diameter were measured by the trained network. For quiescent pores/osteons, the osteon diameter was manually measured, and the wall thickness was calculated as half the difference between osteon diameter and pore diameter.^20^ Even sealed osteonal structures with no central pore, which we rarely observed, were included. Their pore area and diameter were set as 0 while osteon diameters were manually measured. Cortical porosity was derived from the total pore area divided by the total cortical area and multiplied with 100, and cortical thickness was calculated as cortical bone area divided by the length of the periosteal surface.

The reported microstructural parameters included: cortical porosity (%), cortical thickness (μm), pore number (#), mean pore size (μm^2^), mean pore diameter (μm), and pore density (#/mm^2^).

### Histomorphometry—intracortical pore classification

**Figure 1:**
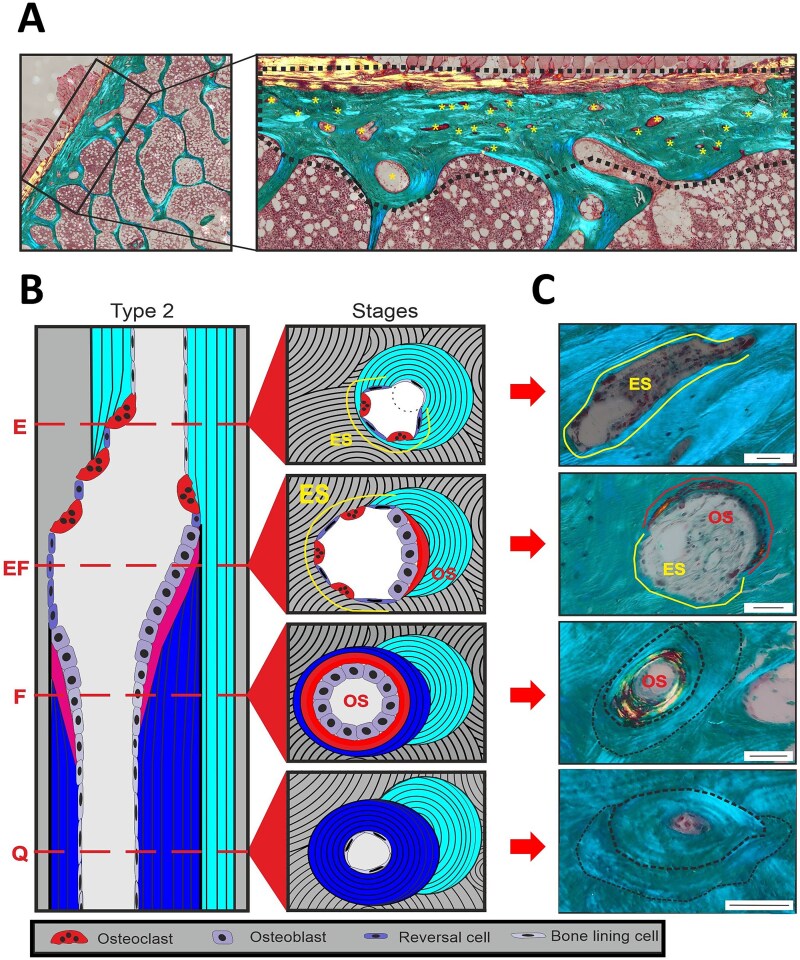
(A) Part of Masson stained iliac crest bone biopsy, with cortical bone outlined by dotted black line and intracortical canals marked by asterisk. (B) Schematic illustrations of type 2 remodeling (remodeling of existing canal) events. Dotted lines mark 4 different stages of remodeling; eroded (E), eroded-formative (EF), formative (F), and quiescent (Q). (B) For each remodeling stage, the intracortical surface represents with distinct features. E-pores present with breaks in the lamellar structures, EF-pores present with both broken lamellar structures and osteoid, F-pores represent with osteoid throughout the pore surface, and Q-pores show no breaks in the lamellar structures. Scale bars = 50 μm.

All intracortical pores were stratified into their respective remodeling stage, based on their bone surface as visualized in Figure 1. Quiescent pores were outlined by surfaces showing no signs of bone erosion (no breaks into the lamellar structures), or bone formation (osteoid). Non-quiescent pores with evidence of active remodeling were classified into either: (1) Eroded pores partly or fully outlined by eroded surfaces with the breakage of the lamellar structures, but with no sign of newly formed osteoid. This indicates that resorption is occurring or has occurred without subsequent initiation of bone formation. (2) Eroded-formative pores outlined by both eroded surfaces and osteoid surfaces, reflecting that transition into bone formation has occurred. (3) Formative pores completely outlined by osteoid surface, reflecting a complete initiation of bone formation.^20^ The reported parameters included: The percentage of pores in a given remodeling stage (% of pores), the percent contribution of pores in a given remodeling stage to the total pore area (% of total pore area), the mean pore area of pores in a given remodeling stage (mean pore area (μm^2^)), and for quiescent pores/osteons the mean pore diameter (μm) and mean wall thickness (μm). All investigations were performed blinded by X.G.D., and cases of doubt were discussed with C.M.A. and T.L.A.

### Histomorphometry—periosteal bone surface

The periosteal bone surfaces were subjected to histomorphometric analysis of eroded (resorptive), osteoid (formative), and quiescent surfaces and reported as the percentage of the total periosteal bone surface. Eroded surfaces were defined as surfaces with resorption cavities with a clear breakage of the lamellar structures.^22^

### Statistics

The original FREEDOM study was initiated and designed by Amgen and a study group of investigators. Amgen holds data about the patients and their treatment. After histological analysis and calculations, statistical analyses were conducted by SH at Amgen, while the other investigators remained blinded. The investigators were not in any way limited in the writing or interpretation of the data by Amgen. The 2- and 3-yr samples were pooled in all analysis, and the 2-yr samples were excluded from patients who had biopsies taken at both time points. Unclassified pores were counted and measured in size but excluded from the analyses of remodeling stages. Comparisons of patient data were performed using the Wilcoxon signed-rank test, whereas comparison of histological data was performed by the 2-sample Wilcoxon rank sum test. The *P*-values below .05 were considered statistically significant.

## Results

In total, 8411 intracortical pores were identified, measured, and classified throughout the 86 analyzed trans iliac bone biopsies. As summarized in Table 1, there were no significant differences in patient characteristics or the number of intracortical pores per biopsy between the denosumab and placebo group at the time of biopsy. In this study, 11% of identified pores were not classified according to their remodeling stage due to insufficient morphological appearance.

**Table 1:** Descriptive data about patients included in this bone biopsy sub-study of FREEDOM and their corresponding biopsies as pooled data from 24- and 36- mo of treatment. Wilcoxon rank sum test was used to test differences between placebo and denosumab, Wilcoxon signed-rank test was used to test differences between baseline and 3-yr.

	Placebo	Denosumab
**Patient data 2 yr**		
**Samples (*n*)**	43	43
**Mean age**	69.95 (±6.46)	72.16 (±4.99)
**T-score, lumbar spine**	−2.89 (±0.50)	−2.86 (±0.55)
**T-score, total hip**	−1.88 (±0.75)	−1.98 (±0.80)
**Patient data 3 yr**		
**Samples (*n*)**	38	40
**T-score, lumbar spine**	−2.90 (±0.57)	−2.27 (±0.63)^a^^,^^b^
**T-score, total hip**	−1.98 (±0.76)	−1.74 (±0.81)^a^
**Biopsy data 2- and 3-yr**		
**Samples (*n*)**	43	43
**No. of pores**	76.72 (±41.86)	71.23 (±44.53)
**No. of unclassified pores**	8.48 (±7.50)	14.72 (±12.69)^b^

a Indicates significant from baseline.

b Indicates significant from placebo.

### The effect of denosumab on cortical microstructure

**Figure 2:**
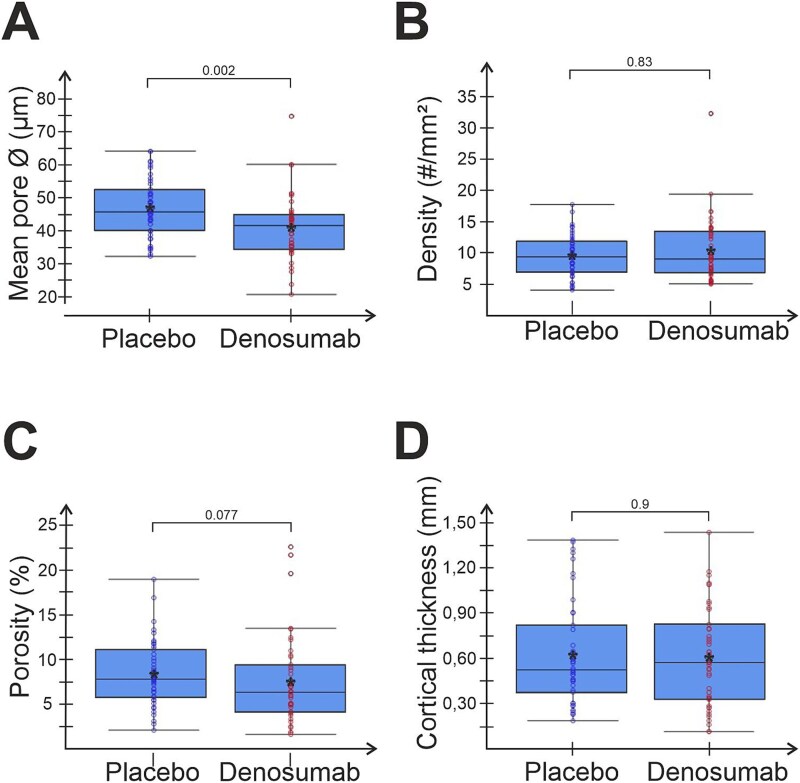
Denosumab might decrease the size of intracortical pores. Graphs showing general macroscopic parameters of analyzed cortical bone and intracortical pores. Each dot represents a biopsy from either denosumab-treated or placebo. Mean within each group is marked by an asterisk. Group comparisons were performed using 2 sample Wilcoxon rank sum test and *P*-values are given in each graph.

The intracortical pore diameter was significantly lower in denosumab- vs placebo-treated patients (*P* = .002, Figure 2A), while the pore density was similar between the treatment groups (*P* = .8, Figure 2B), causing a trend towards lower cortical porosity in the denosumab- vs placebo-treated patients (*P* = .077, Figure 2C). The cortical thickness (*P* = .9, Figure 2D) and mean pore area (*P* = .09, data not shown) were similar between groups. In paired data from patients who had biopsies taken after both 2- and 3-yr, cortical porosity was not decreased either after 2 or 3 yr when compared to placebo. However, cortical porosity was significantly lower after 3-yr of denosumab compared to 2 yr of denosumab (Table S1).

### Denosumab has no effect on quiescent pores and their BMU balance

**Figure 3:**
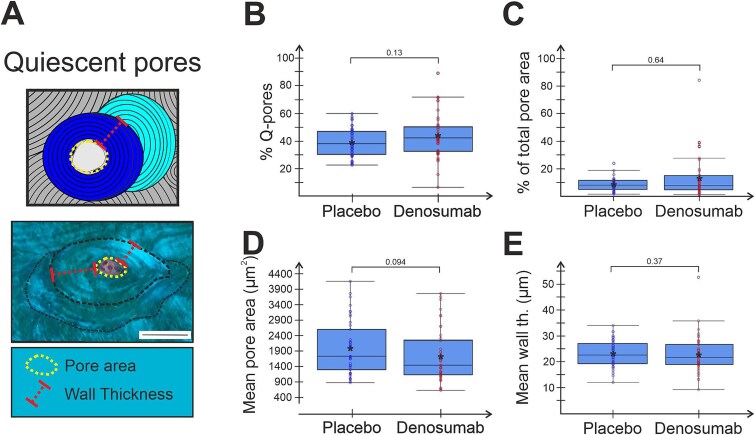
Denosumab treatment does not affect quiescent pores. Illustration of quiescent intracortical pore and obtained measurements (A) and graphs showing prevalence (B), contribution to cortical porosity (C), mean pore area (D), and wall thickness (E) of analyzed quiescent pores. Each dot represents a biopsy from either denosumab-treated or placebo. Mean within each group is marked by an asterisk. Group comparisons were performed using 2 sample Wilcoxon rank sum test and *P*-values are given in each graph.

When analyzing the quiescent intracortical pores/osteons (with completed remodeling events), no statistical difference was observed in either their percentage of all pores (Figure 3B) or percentage contribution to the total pore area (Figure 3C) comparing denosumab- and placebo-treated patients. In addition, the mean area and diameter of quiescent pores were not different (*P* = .094, Figure 3D and not shown) in denosumab compared to placebo treated patients, reflecting an unchanged BMU balance between the magnitude of bone resorption and formation. Finally, the wall thickness of the quiescent pores was not significantly different between patient groups (*P* = .37, Figure 3E), reflecting an unchanged magnitude of bone formed once initiated.

### Formative but not eroded pores are reduced by denosumab treatment

**Figure 4:**
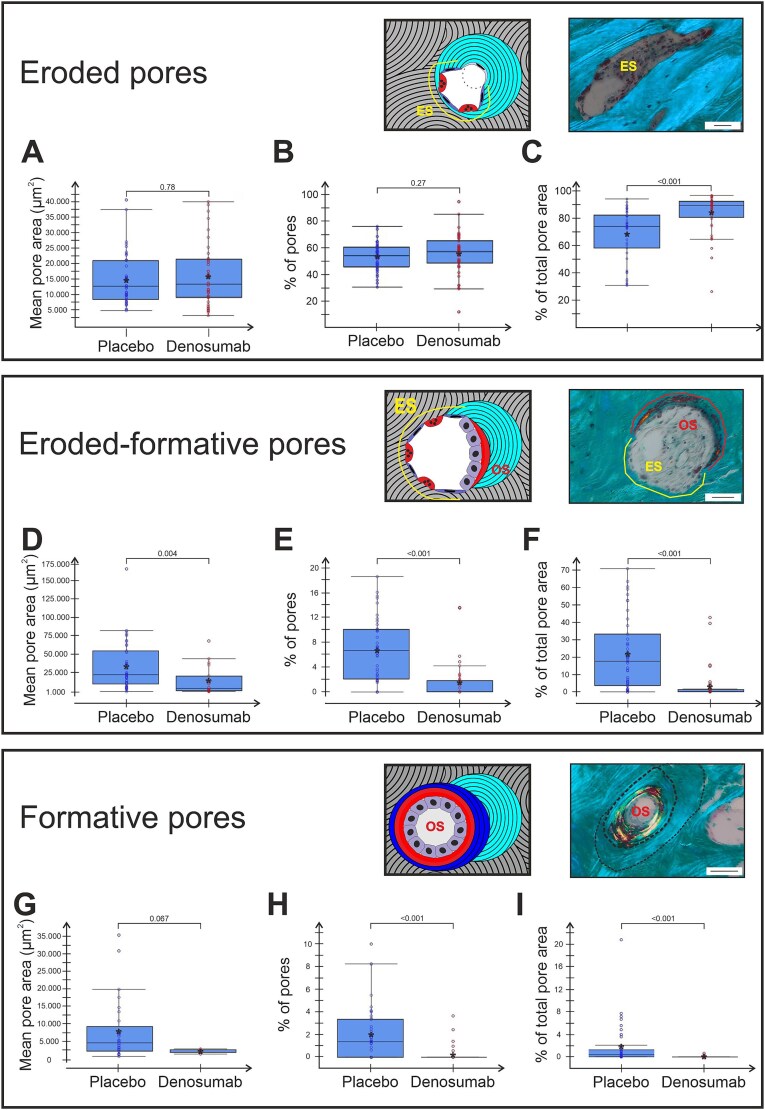
Denosumab treatment affects the prevalence and size of formative pores but not eroded pores. Graphs present the size, prevalence, and contribution to total cortical porosity by each type of non-quiescent pore. Each dot represents a biopsy from either denosumab-treated or placebo. Mean within each group is marked by an asterisk. Group comparisons were performed using 2 sample Wilcoxon rank sum test and *P*-values are given in each graph.

Osteoclasts are considered depleted from the bone surfaces by denosumab, thus expectedly decreasing the resorption activity. Nevertheless, we did not observe a decrease in either the eroded pores mean pore area (Figure 4A) or the percentage of all pores (Figure 4B) in denosumab vs placebo treated patients. However, their relative contribution to the total pore area (% of total pore area) was significantly higher with denosumab than placebo (*P* < .001, Figure 4C). This could be explained by a significantly lowered prevalence of eroded-formative (*P* < .01, Figure 4E) and formative pores (*P* < .001, Figure 4H). Furthermore, eroded-formative pores were significantly smaller (mean pore area) in the denosumab group compared to placebo (*P* = .004, Figure 4D), thereby contributing less to the total pore area (% of total pore area). Also note, only 4 biopsies from the denosumab group presented with any formative pores, which further underscores the lack of formation activity.

### Denosumab leads to decreased periosteal formation

**Figure 5:**
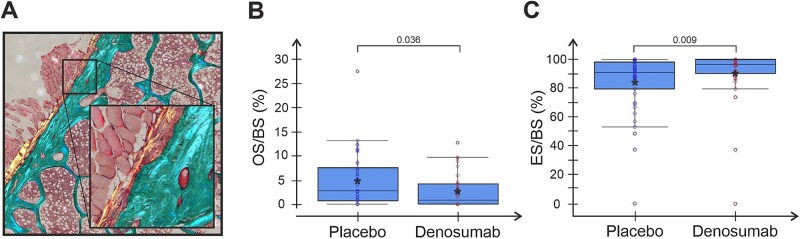
Denosumab treatment significantly alters periosteal surfaces by increasing erosion and reducing formation. Graphs showing percentage of periosteal surfaces with formation (A) or erosion (B). Each dot represents a biopsy from either denosumab-treated patients or placebo. Mean within each group is marked by an asterisk. Group comparisons were performed using 2 sample Wilcoxon rank sum test and *P*-values are given in each graph.

A proposed explanation of the continuous increase in BMD observed with denosumab treatment is an increase in periosteal bone formation without prior resorption (modeling-based bone formation), which has been described in the femoral bone of monkeys and the human femur neck.^18^^,^^19^ However, in this study we observed a significantly lower percentage (2.6 ± 3.5% vs. 4.8 ± 5.5%) of the periosteal surface undergoing bone formation (osteoid surfaces per bone surface, OS/BS) in denosumab vs placebo treated patients (*P* = .036, Figure 5B). Instead, in patients receiving denosumab, almost all their periosteal surface (90.5 ± 19.9%) was eroded with a significantly higher eroded surface per bone surface (ES/BS) in the denosumab vs placebo group (Figure 5C).

## Discussion

Studies on the cortical microstructural effects of denosumab are scarce, and mainly based on histomorphometry, HR-pQCT, and BMD yielding variable results among studies and skeletal sites investigated. From a cortical thickness perspective, HR-pQCT studies have shown a decreased cortical volume in the distal radius after 12 mo of treatment compared to controls, but an increased trabecular bone volume,^23^ and an increased cortical thickness in the radius and tibia after 24 mo of denosumab treatment.^24^ Structural analysis based on DXA showed an increased cortical thickness in the femoral intertrochanter and shaft of patients treated with denosumab for 24-mo,^25^ 36 mo,^26^ and 30-48 mo.^27^ On the other hand, bone histomorphometry studies of trans iliac bone biopsies showed no effect of denosumab on the cortical thickness after either 2 or 3 yr of treatment,^16^^,^^17^ in line with this study.

When considering cortical porosity, quantitative analysis of CT scans of the femoral shaft has shown significant reductions in cortical porosity with 3 yr of denosumab treatment compared to placebo,^15^ and HR-pQCT has shown reduced porosity in the radius after 1 yr of denosumab treatment^23^ or no effect in the radius and tibia over 2 yr.^24^ The resolution of CT scanners usually hinders the detection of pores with a diameter below approximately 20-40 μm for μCT and 80-190 μm for HR-pQCT,^28^ whereas histomorphometric studies allow the detection of even sealed pores, thus including all pores. Our histomorphometric analysis of the iliac crest biopsies showed a significant reduction in the mean pore size, but no change in the pore density, causing a trend towards reduced cortical porosity. In contrast to previous studies on biopsies from the FREEDOM study,^16^^,^^17^ also showing a slight reduction in the cortical porosity after 2 yr but not after 3 yr, our study pooled the 2- and 3-yr treatment groups to increase the power.

The activation of bone resorption and its transition to bone formation are key steps in the coupled bone remodeling process. Insufficient coupling contributes to bone loss during aging and osteoporosis.^6^^,^^10^^,^^20^ Here, the activation of bone resorption with a delayed or absent transition to bone formation results in an accumulation of enlarged pores with eroded surfaces causing bone loss.^20^ Denosumab is a potent anti-resorptive therapy that inhibits osteoclast differentiation and fusion, and thereby the activation of bone resorption and the subsequent remodeling process. Furthermore, ongoing resorption is also inhibited.^29^ Accordingly, we observe a higher contribution of quiescent pores (no remodeling activity) to the total pore area upon denosumab treatment. But osteoclasts are also critical for the eroded surfaces or pores transition to bone formation.^10^ During the reversal-resorption phase of the bone remodeling process, osteoclasts are intermixed with osteoprogenitors (reversal cells) on the eroded surfaces, which progress to bone formation when the osteoprogenitors reach a critical density and differentiate into bone-forming osteoblasts.^9^^,^^22^^,^^30^ The osteoprogenitors on eroded surfaces are gradually recruited and differentiated in response to local factors, which are most likely osteoclastic coupling factors. Such coupling factors can be bone matrix-derived factors released during resorption, membrane-bound factors on osteoclasts, or factors secreted by osteoclasts.^31–33^ Dependency on these coupling factors makes osteoclasts and their resorption a prerequisite for the initiation of bone formation, but osteoclasts are depleted with denosumab-treatment, thereby suppressing known coupling factors.^34^ In line with this, our study shows that the prevalence of eroded pores is unchanged upon denosumab treatment, while the prevalence of the subsequent formative pores is drastically reduced as they are gradually completed and thereby converted to quiescent pores. Besides the finalization of initiated formative pores, it is possible that some pores having both eroded and formative surfaces fail to go into complete formation. In this case, the osteoid part of eroded-formative pores will gradually become mineralized and thereby quiescent whereas the eroded part will remain eroded. The transformation of eroded-formative pores to either eroded or quiescent pores, significantly increases the contribution of eroded pores to cortical porosity, because of the size difference between stages. Eroded-formative pores are on average twice as big as eroded pores and 14-fold bigger than quiescent pores. This size difference allows eroded pores to contribute more to total cortical porosity when eroded-formative pores terminate and become quiescent or transform into eroded pores. This hypothesis seems appealing, as we during our analysis observed pores with a scattered pattern of osteoid even though part of the surfaces were still eroded (as seen in the eroded-formative pore Figure 1B), however this was not quantified. An additional increase in contribution of eroded pores to porosity may result from the previously reported short alleviation of resorptive suppression by the end of each dosage interval.^35^^,^^36^ Temporary osteoclast activation in the end of each dosage interval would be enough to allow some erosion, but we do not know whether this occurs in all patients. Furthermore, the effect of anti-RANKL (denosumab) treatment seems to vary between the trabecular and cortical envelope.^37^ Hence, the alleviation of resorptive suppression may have different effects on the trabecular vs cortical envelope.

These data imply that denosumab arrests the eroded pores in the status they are at the time of denosumab administration. Hence, at the time of the first denosumab administration, eroded pores remain eroded and do not progress into bone formation. One should thus be aware that the eroded pores identified in denosumab-treated samples probably are arrested pores due to resorption prior to denosumab administration. Thus, in this study we are discussing resorption, which was initiated 2- to 3 yr before the biopsies were taken—an aspect that is often overlooked.

The reduced prevalence of formative pores upon denosumab treatment, drastically changes the remodeling stage of those pores contributing the most to the overall cortical porosity. This is further enhanced by the reduced size of formative and eroded-formative pores, leading to a highly significant reduction in the formative and eroded-formative contribution to the overall porosity. Accordingly, the eroded pores end up having a significantly higher contribution to the overall porosity, even though their prevalence and size are unchanged. These changes in the underlying pores contributing to the cortical porosity are overlooked in classical surface-based histomorphometry and CT-based studies of the cortical effect of denosumab, where the remodeling stage is not linked with the observed cortical porosity. A similar accumulation of eroded pores with no transition to formation has been reported in a patient treated with the bisphosphonate alendronate,^38^ and observed in a patient experiencing an atypical femur fracture after 5 yr of bisphosphonate treatment.^39^ Whether this gradual accumulation of eroded pores potentially contributes to the rare atypical cortical femur fractures occasionally observed in long-term users of anti-resorptive treatment remains to be investigated.^3^^,^^36^^,^^40^

Cortical histomorphometry of quiescent osteons provides a unique perspective into the effect of denosumab on the remodeling balance at the individual BMU level, also called the BMU balance.^7^ Denosumab treatment had no effect on either the wall thickness or the pore area. This reflects the magnitude of bone formed prior and during the early denosumab treatment as well as the BMU balance between the magnitude of resorption and formation. In other words, denosumab had no effect on the bone formation once initiated.

**Figure 6:**
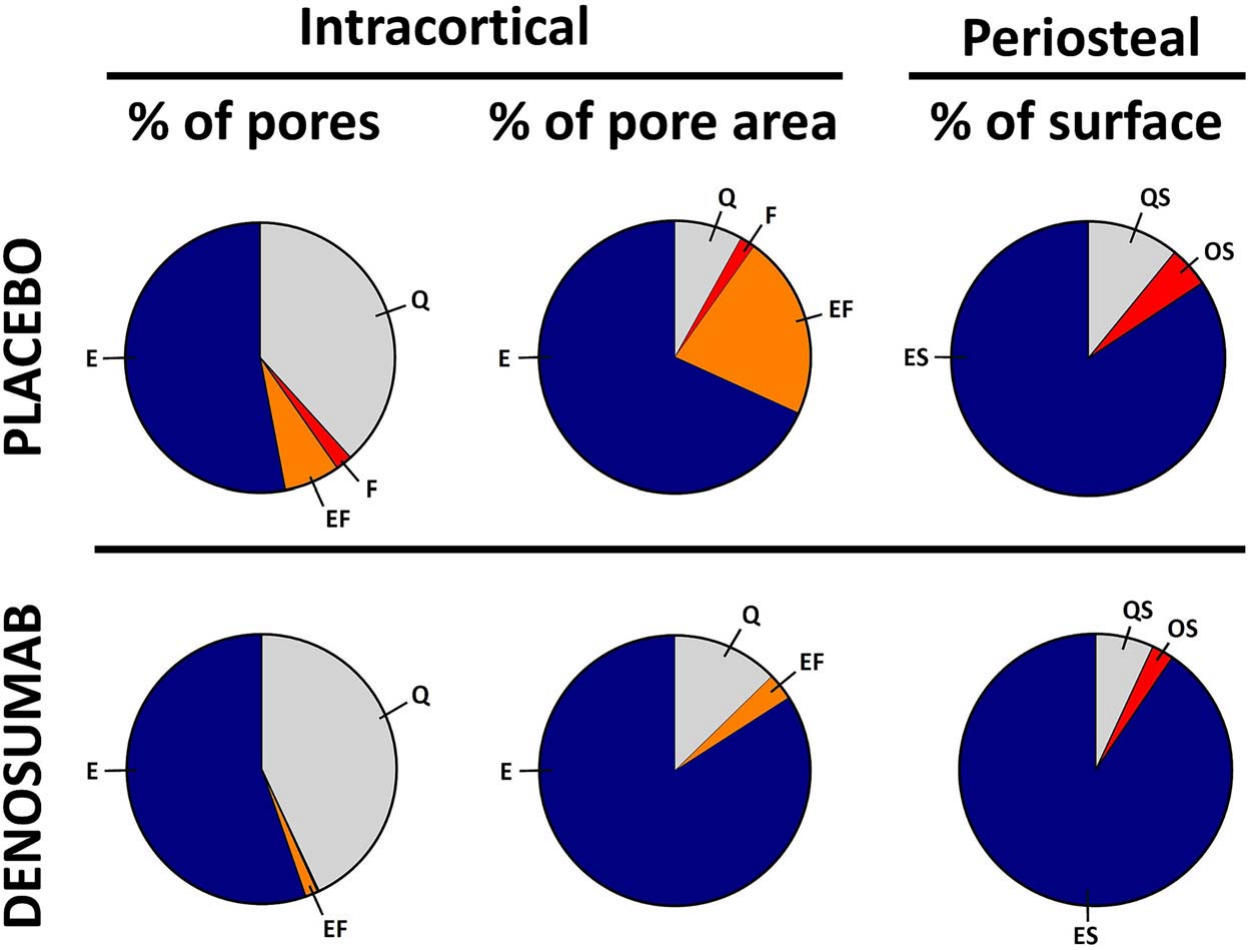
Visualization of the cortical and periosteal effects of denosumab. The left-side diagrams show the percentwise distribution of remodeling stages in placebo vs denosumab. The diagrams in the middle show how each remodeling stage contributes to total cortical porosity. The right-side diagrams show the contribution of quiescent (QS), formative (OS), and eroded (ES) surfaces on the periosteal side of biopsies.

Investigations of long-term effects of denosumab have shown continued reductions in bone formation markers for up to 10 yr.^36^^,^^41^ Although markers of bone formation and resorption remained suppressed, a continued increase in BMD at other skeletal sites has been reported.^36^^,^^41^ This continued increase in BMD has been ascribed to an increased secondary mineralization,^42^ as well as osteoclast-independent modeling-based bone formation on the periosteal surface of cortical bone.^18^^,^^19^ We assessed the percentage of periosteal osteoid surfaces and observed a significant reduction in these periosteal bone forming surfaces, as summarized in Figure 6. Iliac crest is undoubtedly not subjected to the same loading conditions as the femur but remains loaded by the Iliacus muscles. This difference in loading may explain why we were unable to observe a maintained bone formation on the periosteal surface, contrasting to the previously observed modeling-based formation on the periosteal surface of the femur from monkeys and 4 humans treated with denosumab.^19^

In contrast, the reported reduction in bone formation combined with a reduction in new resorption supports the hypothesis that part of the enhanced BMD is caused by an extended secondary mineralization caused by the suppressed bone turnover.

### Limitations

This study was conducted in trans iliac biopsies from the previous FREEDOM clinical trial. In each treatment group 43 biopsies were available. Notably, 25 patients had biopsies taken after both 2 and 3 yr. Since the biopsies were not collected in a new study, a power analysis was not performed prior to the study and the design and collection of biopsies might not be sufficient to detect small changes associated with treatment. To increase the statistical power, 2- and 3-yr samples were pooled, however excluding biopsies from the second year from patients who had biopsies taken at both time points. Biopsies were collected 56 d before their last dosage of denosumab, making it impossible to address whether the bone resorption recovers towards the end of the 6-mo dosing,^16^ as some studies have indicated a recovery of the bone resorption by the end of each dosage interval.^36^^,^^43^ Whether bone remodeling is significantly different at individual time points after dosing is thus not assessed.

The analysis was restricted to iliac crest biopsies from postmenopausal women. Whether findings are applicable to other skeletal sites, males, or patients with other bone-related diseases is not examined. Importantly, this investigation focuses on the pooled effects of denosumab following 2- and 3-yr of treatment to increase the power of the analysis but may hide any differences between 2 and 3 yr of treatment (see Table S1).

## Conclusion

This study demonstrates that the transition from eroded to formative pores is inhibited by denosumab in osteoporotic patients, reflecting an arrest in intracortical bone remodeling events in the reversal-resorption phase. Moreover, the generation of new remodeling events is nearly ablated, implying that the observed eroded pores largely reflect the eroded pores present prior to treatment initiation. Pores undergoing formation at the time of treatment initiation are completely refilled with no effect on the magnitude of bone formed. We hypothesize that the arrest of bone remodeling events in the reversal-resorption phase can be ascribed to the absence of osteoclast-derived coupling factors, driving the expansion and maturation of the osteoprogenitors that are critical for the transition to formation. Therefore, the continued increases in BMD measured in clinical trials cannot be explained by a complete closure of all previous bone remodeling events. Whether this increase is ascribed to modeling-based bone formation on the periosteal surfaces of the cortical bone or continued secondary mineralization of old bone remains to be investigated.

## Supplementary Material

Supplementary_ziaf181

## Supplementary material

Supplementary material is available at *JBMR Plus* online. Qualified researchers may request data from Amgen clinical studies. Complete details are available at the following: http://www.amgen.com/datasharing
