# Supplementary material for: Denosumab treatment of osteoporotic women arrests cortical bone remodeling events at the reversal-resorption phase but does not affect ongoing bone formation
Source: JBMR Plus. 2025 Nov 22;10(1):ziaf181. doi: 10.1093/jbmrpl/ziaf181 (PMC12771366; doi:10.1093/jbmrpl/ziaf181)
Supplement: Supplementary_ziaf181 [file supplementary_ziaf181.pdf]

Supplementary figure 1

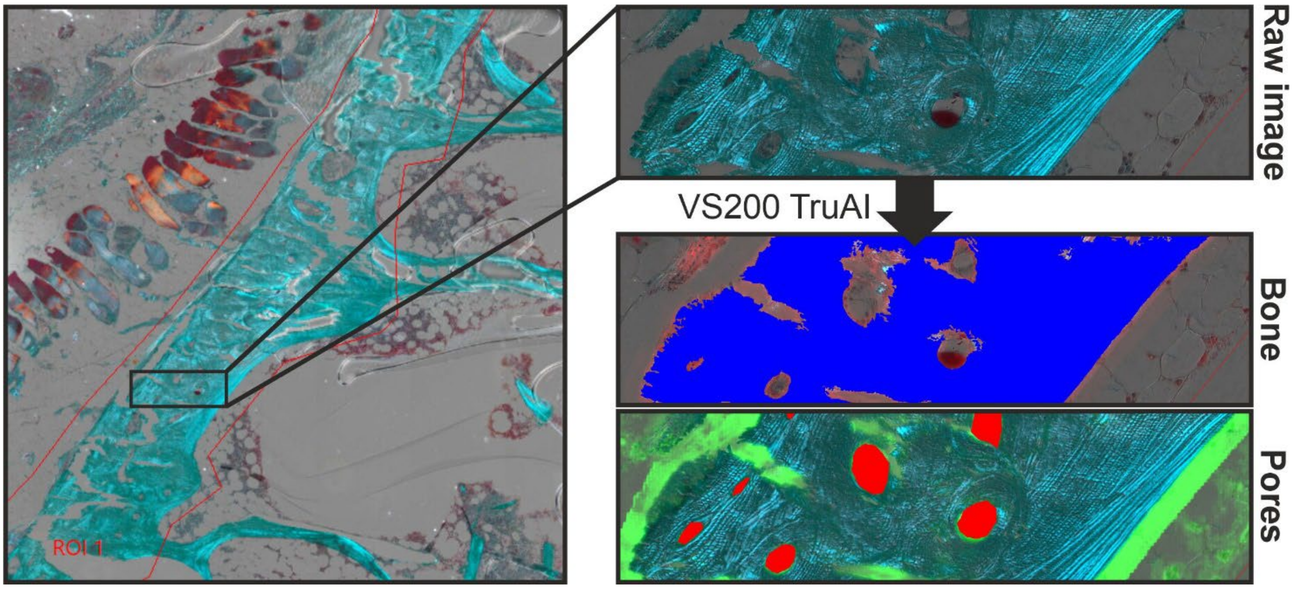

Supplementary figure 1
Images illustrating the output of VS200 TruAI software for identification of cortical bone and intracortical pores. Left: Scanned image of one cortex with ROI marked with red line. Right: Zoom showing the raw scan and segmented images below black arrow with identified cortical bone (blue) and identified intracortical pores (red). The software succesfully excluded tissue artifacts from the recognized cortical bone, whereas the manual check optimized identification of intracortical pores and enabled exclusion of these tissue artifacts.

parameters	Paired biopsies from Denosumab treated patients					Denosumab versus placebo	
	Denosumab – month 24		Denosumab – month 36			Month 24	Month 36
	n	Mean (SD)	n	Mean (SD)	*p*	*p*	*p*
Cortical microstructure							
Co.Po (%)	6	10.3 (±4.9)	6	6.3 (±3.2)	**0.031**	0.097	0.58
Co.Th (µm)	6	771 (±364)	6	812 (±187)	0.84	0.74	0.40
Pore density (#/mm^2)	6	10.4 (±4.6)	6	8.3 (±2.5)	0.44	0.80	0.91
Mean Pore Area (µm^2)	6	14281 (±12018)	6	8403 (±3750)	0.44	0.25	0.80
Mean Pore Diameter (µm)	6	51.2 (±10.1)	6	43.0 (±7.7)	0.16	0.11	0.63
Periostal surfaces							
ES/BS (%)	5	83.7 (±18.3)	5	93.2 (±8.5)	0.38	0.76	0.25
OS/BS (%)	5	2.5 (±2.7)	5	1.2 (±1.8)	0.25	0.22	0.10
Eroded pores							
% of all pores	6	54.2 (±12.2)	6	49.9 (±14.2)	0.69	0.40	0.44
% of total area	6	76.3 (±27.7)	6	83.2 (±15.8)	0.56	0.051*	0.29
Mean area (µm^2)	6	248678 (±23510)	6	15148 (±5209)	0.44	0.15	0.74
Eroded-formative pores							
% of all pores	6	5.8 (±7.9)	6	1.1 (±1.9)	0.25	0.17	**0.008**
% of total area	6	16.3 (±26.4)	6	0.3 (±0.6)	0.25	**0.05**	**0.002**
Mean area (µm^2)	1	6334 (±0.0)	1	1837 (±0.0)	1.00	0.19	0.19
Formative pores							
% of all pores	6	0.1 (±0.3)	6	0.0 (±0.0)	1.00	**0.037**	**0.018**
% of total area	6	0.0 (±0.0)	6	0.0 (±0.0)	1.00	**0.037**	**0.018**
Mean area (µm^2)	0	NA	0	NA	NA	NA	NA
Quiescent pores							
% of all pores	6	39.9 (±18.8)	6	49.1 (±14.2)	0.44	0.71	0.17
% of total area	6	7.4 (±3.4)	6	16.5 (±16.1)	0.44	0.85	0.44
Mean area (µm^2)	6	2561 (±972)	6	2099 (±922)	0.56	**0.004**	0.36
Mean wall thickness (µm)	6	23.0 (±3.7)	6	56.9 (±6.1)	0.44	0.4	**0.071**

Supplementary table 1

24 patients in the FREEDOM bone biopsy sub-study had biopsies taken after both 2 and 3 years. In this table, denosumab treated patients (n = 6) were compared pairwise from 2 to 3 years of denosumab treatment. Furthermore, denosumab treated biopsies were compared to placebo (n = 16) at both time points. Bold font indicates significant p-value, * indicates a near-significant p-value. Statistics: Wilcoxon signed-rank test.
